# INTEGRATE-Vis: a tool for comprehensive gene fusion visualization

**DOI:** 10.1038/s41598-017-18257-2

**Published:** 2017-12-19

**Authors:** Jin Zhang, Teng Gao, Christopher A. Maher

**Affiliations:** 1 Department of Radiation Oncology, Washington University School of Medicine, St. Louis, Missouri 63110 USA; 2 McDonnell Genome Institute, Washington University School of Medicine, St. Louis, Missouri 63110 USA; 3 Department of Internal Medicine, Division of Oncology, Washington University School of Medicine, St. Louis, Missouri 63110 USA; 4 Siteman Cancer Center, Washington University School of Medicine, St. Louis, Missouri 63110 USA; 5 Department of Computer Science and Engineering, Washington University, St. Louis, Missouri 63105 USA; 6 Department of Biomedical Engineering, Washington University, St. Louis, Missouri 63105 USA

## Abstract

Despite the increasing quantity of tools for accurately predicting gene fusion candidates from sequencing data, we are still faced with the critical challenge of visualizing the corresponding gene fusion products to infer their biological consequence (i.e. novel protein and increased gene expression). This is currently accomplished by manually inspecting and inferring the biological consequence of top scoring gene fusion candidates. This labor-intensive process could be made easier by automating the annotation of gene fusion products and generating easily interpretable visualizations. We developed a gene fusion visualization tool, called INTEGRATE-Vis, that generates comprehensive, highly customizable, publication-quality graphics focused on annotating each gene fusion at the transcript- and protein-level and assessing expression within an individual sample or across a patient cohort. INTEGRATE-Vis is the first comprehensive gene fusion visualization tool to help a user infer the potential consequence of a gene fusion event. It has potential utility in both research and clinical settings. INTEGRATE-Vis is available at https://github.com/ChrisMaherLab/INTEGRATE-Vis.

## Introduction

Gene fusions have served as highly specific diagnostic markers, prognostic indicators and therapeutic targets^1^. High throughput transcriptome sequencing (RNA-Seq) has accelerated our ability to discover expressed gene fusions^2^. While recent tools, such as INTEGRATE^3^, are highly sensitive and specific, we are still faced with the critical challenge of ensuring that a casual gene fusion is not only detected, but that it can be prioritized accordingly amongst passenger events. This is currently accomplished by manually inspecting and inferring the biological consequence (i.e., generation of a novel protein, altered expression levels) of top scoring gene fusion candidates. The labor-intensive process could be made easier and more precise by automating the annotation of gene fusion transcripts and proteins and generating easily interpretable visualizations. Currently gene fusion visualization approaches either rely on CIRCOS to highlight the genomic locations of the gene fusion partners^4^, IGV for assessing sequence coverage at fusion junctions^5^, or splicing graphs to observe the exons involved in a gene fusion^6^. Individually each of these methods is insufficient for inferring the consequences of the gene fusion on expression or the corresponding protein product. To address these limitations, we developed a tool, INTEGRATE-Vis, which generates multiple visualizations for annotating each gene fusion, at the transcript- and protein-level, and assessing gene expression within an individual sample or across a cohort.

## Results

**Figure 1:**
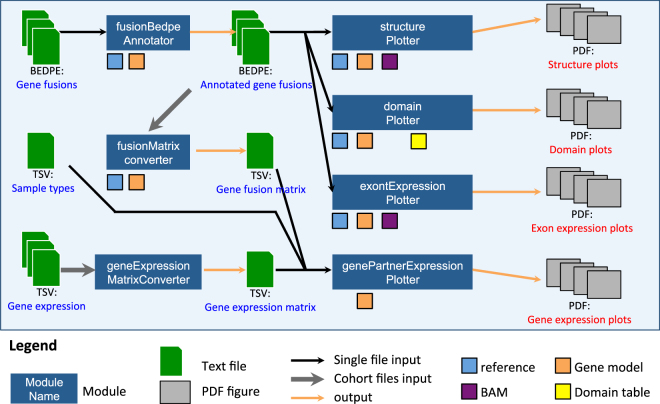
Overview of the INTEGRATE-Vis pipeline. INTEGRATE-Vis contains four major modules to plot four types of figures for gene fusions: isoform structure, domain, exon expression, and gene partner expression. The first three modules are for individual samples, and the last module is for cohort data. Only minimal inputs in standard formats, including BEDPE, TSV, FASTA, BAM, and GTF are needed to make the plots.

**Figure 2:**
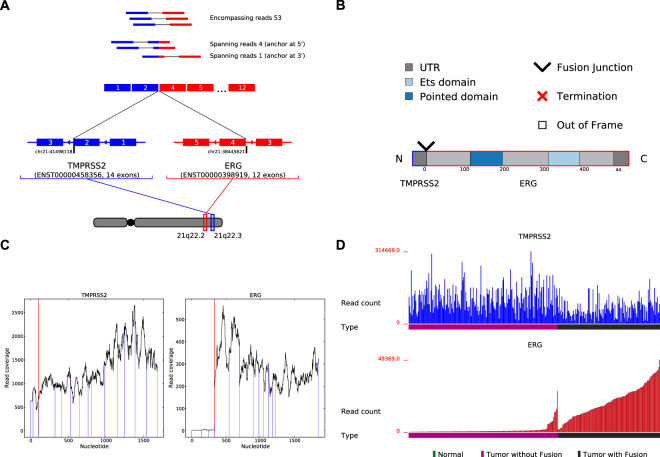
INTEGRATE-Vis output illustrated using the *TMPRSS2-ERG* gene fusion in prostate cancer. INTEGRATE-Vis outputs four visualizations including: (**A**) gene fusion transcript isoforms, (**B**) the predicted protein structure of the gene fusion, (**C**) RNA-Seq read coverage across each gene fusion partner to reveal changes in exon expression (A red line is plotted at the fusion junctions at both gene partners. Exon boundaries are represented by blue lines. A marked expression change occurs between exons 3 and 4 of *ERG*.), and (**D**) expression of each gene fusion partner across the TCGA PRAD cohort. Blue is used to represent supporting reads, exons, transcript, and genomic locations for the 5′ gene partner (*TMPRSS2*), while red is for those of the 3′ gene partner (*ERG*).

INTEGRATE-Vis pipeline (Fig. 1) generates four types of figures, created manually in previous publications, to provide easy-to-interpret visualizations of gene fusion predictions^3,7–10^. To illustrate how INTEGRATE-Vis works we focused on a prostate cancer patient harboring the most prevalent gene fusion, *TMPRSS2-ERG*, that results in the marked increase in the expression of the oncogenic transcription factor *ERG* (Fig. 2). Panels A through D of Fig. 2 correspond to structure plot, domain plot, exon expression plot, and gene expression plot outputs described in Fig. 1, respectively.

First, in our structure plot we show the predicted gene fusion transcript structure highlighting sequence reads that encompass and span the fusion junction (Fig. 2A). Both *TMPRSS2* and *ERG* are on the reverse strand of chromosome 22 in two consecutive cytogenetic bands. A genomic deletion between the upstream gene, *TMPRSS2*, and the downstream partner, *ERG*, generates the gene fusion event. By default, INTEGRATE-Vis constructs a gene fusion transcript isoform using the most prevalent transcript isoform of the gene partners. Alternatively, a user can designate specific transcript isoforms to display in the reconstructed gene fusion transcript. As shown in Fig. 2A, a 14-exon isoform (ENST00000458356) of *TMPRSS2* and a 12-exon isoform (ENST00000398919) of *ERG* were used for visualization. The fusion junction is located at the second exon of ENST00000458356 and the fourth exon of ENST00000398919. The gene fusion transcript is shown in the upper panel with the corresponding supporting reads to infer the expression level. To conserve space, INTEGRATE-Vis displays 1, 2, or 3 supporting reads to illustrate 1, 2–10, or >10 supporting sequence reads, respectively.

Second, to predict the potential functional consequences of a gene fusion INTEGRATE-Vis generates a domain plot to translate the fusion transcript and displays the corresponding protein domains (Fig. 2B). The protein product from the 5′ gene partner (i.e. *TMPRSS2*) is plotted on the left and the protein product from the 3′ gene partner (i.e. *ERG*) is plotted on the right. This is also indicated by the annotation of N and C for N- and C- terminuses (Fig. 2B). As shown in Fig. 2B, the in-frame gene fusion protein product is comprised of a small 5′ regulatory region of *TMPRSS2* and the majority of *ERG*. This includes both the ETS domain and Pointed domain of the *ERG* gene. As shown in Figure S1, for out-of-frame gene fusion transcripts, the 3′ end is represented by a white box and a red cross is used to represent the translation termination site.

Third, to determine whether the gene fusion increases the expression of the 3′ partner (i.e., oncogene) or decreases the expression of the 5′ partner (i.e., tumor suppressor), INTEGRATE-Vis generates an exon expression plot. This displays read coverage for the exon-level expression for each gene involved in the fusion (Fig. 2C). As shown in Fig. 2C, the *ERG* exons involved in the gene fusion (exons 4 through 12) have significantly higher read coverage compared to the exons that are not included in the gene fusion (exons 1 through 3). Notably, INTEGRATE-Vis automatically selects the scales for the y- and x-axes to show the ranges of read coverage for each gene partner, although they can be adjusted based on user-defined input (Figure S2).

Fourth, to determine if the sample harboring the gene fusion results in a unique expression change relative to a cohort of samples (e.g., prostate cancer patients lacking the gene fusion), INTEGRATE-Vis generates a gene expression plot. This outputs a bar plot of the expression level for both genes involved in the fusion across a patient cohort (Fig. 2D). For example, Fig. 2D highlights the difference in *ERG* expression levels in patients lacking the *TMPRSS2-ERG* gene fusion with patients harboring the *TMPRSS2-* *ERG* gene fusion. In contrast, expression levels of *TMPRSS2* are not different between patients with or without the gene fusion. In addition to determining if the gene fusion alters the expression of the 5′ or 3′ gene, this visualization can also identify additional patients that may also harbor gene fusions producing similar expression consequences.

While we have demonstrated the utility of INTEGRATE-Vis using the most prevalent *TMPRSS2-ERG* isoform, INTEGRATE-Vis automatically generates plots for all predicted gene fusion isoforms. INTEGRATE-Vis has been implemented with reasonable default parameters to help best interpret the functions of the gene fusion products. It also provided ample options to enhance user-friendliness (Figures S2 and S3). INTEGRATE-Vis executes efficiently; figure generation takes a few seconds (Figure S4).

## Discussion

Overall, we developed the first comprehensive gene fusion visualization tool, INTEGRATE-Vis, which generates publication-quality graphics to help a user infer the potential consequence of a gene fusion event. We have implemented INTEGRATE-Vis to utilize standardized input files, including the SMC-RNA BEDPE format for gene fusion predictions, therefore making it widely accessible to the larger research community independent of the gene fusion discovery tool being used.

## Methods

The INTEGRATE-Vis pipeline was implemented in Python and C++, and requires a minimal set of dependencies (CMake, GCC, Matplotlib, and gtfToGenePred) to install and execute. The input into INTEGRATE-Vis includes a list of gene fusion candidates in a standard BEDPE format as well as other common standardized inputs (i.e. FASTA, GTF) including a reference genome and gene models. 333 BEDPE files can be downloaded from https://github.com/ChrisMaherLab/INTEGRATE-Vis, including gene fusions previously discovered^10^. Additional input files in TSV format (i.e. a protein domain table and an ideogram table for cytogenetic bands) and the commands to generate these TSV files are all included at https://github.com/ChrisMaherLab/INTEGRATE-Vis. Read counts for the samples were calculated using FeatureCounts^11^. INTEGRATE-Vis performs a series of annotation and calculation steps before generating figures summarizing the gene fusion in PDF format (Fig. 1).

### Availability and requirements

The INTEGRATE-Vis pipeline has been tested using Python version 2.7 and requires CMake, GCC, Matplotlib, and gtfToGenePred to install and execute. It is available from https://github.com/ChrisMaherLab/INTEGRATE-Vis, which also contains instructions and links for downloading required tools or packages, step-by-step instructions of installing INTEGRATE-Vis pipeline, and sample command lines of executing INTEGRATE-Vis from either BEDPE files or raw RNA-seq reads. Raw sequence reads of TCGA PRAD cohort can be downloaded from Genomic Data Commons (https://gdc.cancer.gov).

## Electronic supplementary material

Supplementary Figures
