# Supplementary material for: INTEGRATE-Vis: a tool for comprehensive gene fusion visualization
Source: Sci Rep. 2017 Dec 19;7:17808. doi: 10.1038/s41598-017-18257-2 (PMC5736641; doi:10.1038/s41598-017-18257-2)
Supplement: Supplementary file 1 — Supplementary Figures [file 41598_2017_18257_MOESM1_ESM.pdf]

# Supplementary Figures

## INTEGRATE-Vis: a tool for comprehensive gene fusion visualization

Jin Zhang[1], Teng Gao[2,5], and Christopher A. Maher[2,3,4,6*],

[1]Department of Radiation Oncology, Washington University School of Medicine, St. Louis, Missouri 63110, USA.
[2]McDonnell Genome Institute, Washington University School of Medicine, St. Louis, Missouri 63110, USA.
[3]Department of Internal Medicine, Division of Oncology, Washington University School of Medicine, St. Louis, Missouri 63110, USA.
[4]Siteman Cancer Center, Washington University School of Medicine, St. Louis, Missouri 63110, USA.
[5]Department of Computer Science and Engineering, Washington University, St. Louis, Missouri 63105, USA.
[6]Department of Biomedical Engineering, Washington University, St. Louis, Missouri 63105, USA.

*To whom correspondence should be addressed.
Contact christophermaher@wustl.edu

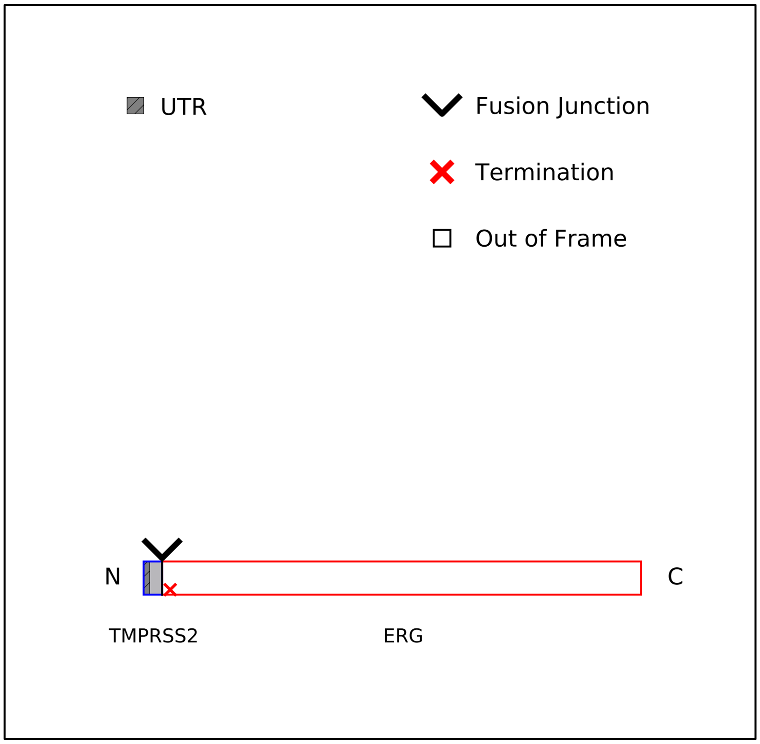


**Figure S1. A gene fusion isoform that produces an early termination site.** This domain figure corresponds to an alternative gene fusion isoform different from the one shown in Figure 2, but is discovered in the same patient.

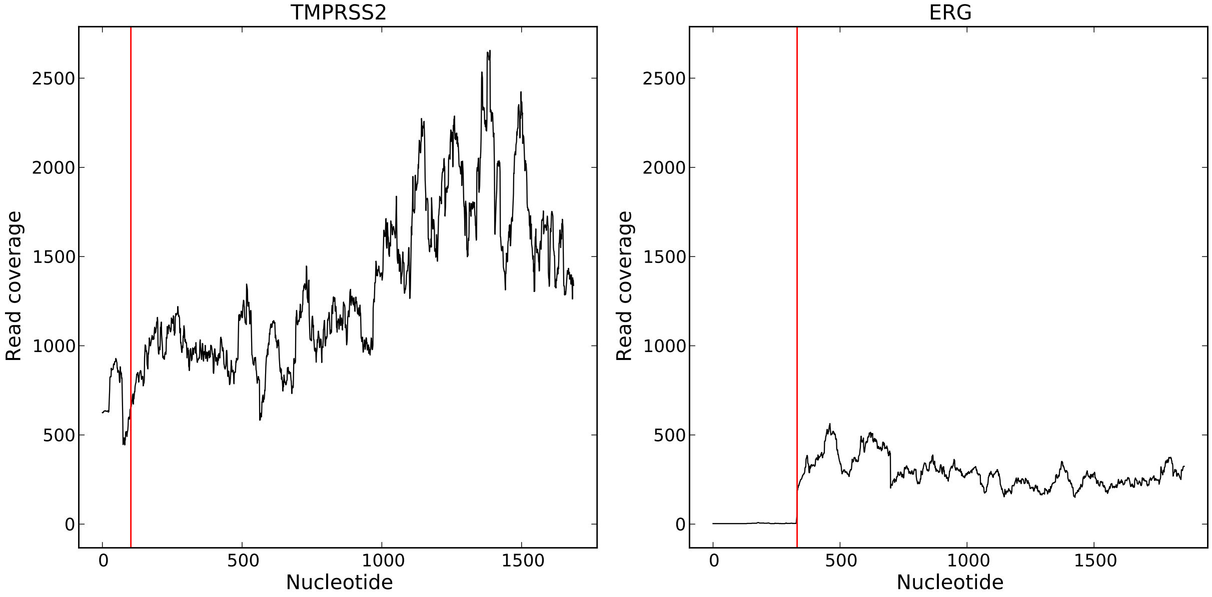


**Figure S2. Users can control the scales of the exon expression figure.** This figure shows the same plot as Figure 2C, except that the y-axis is scaled with the same value for the left and right panels, and the lines for exon boundaries are removed. User can also control the scales for both the y- and x-axes of both the left and right panels independently.

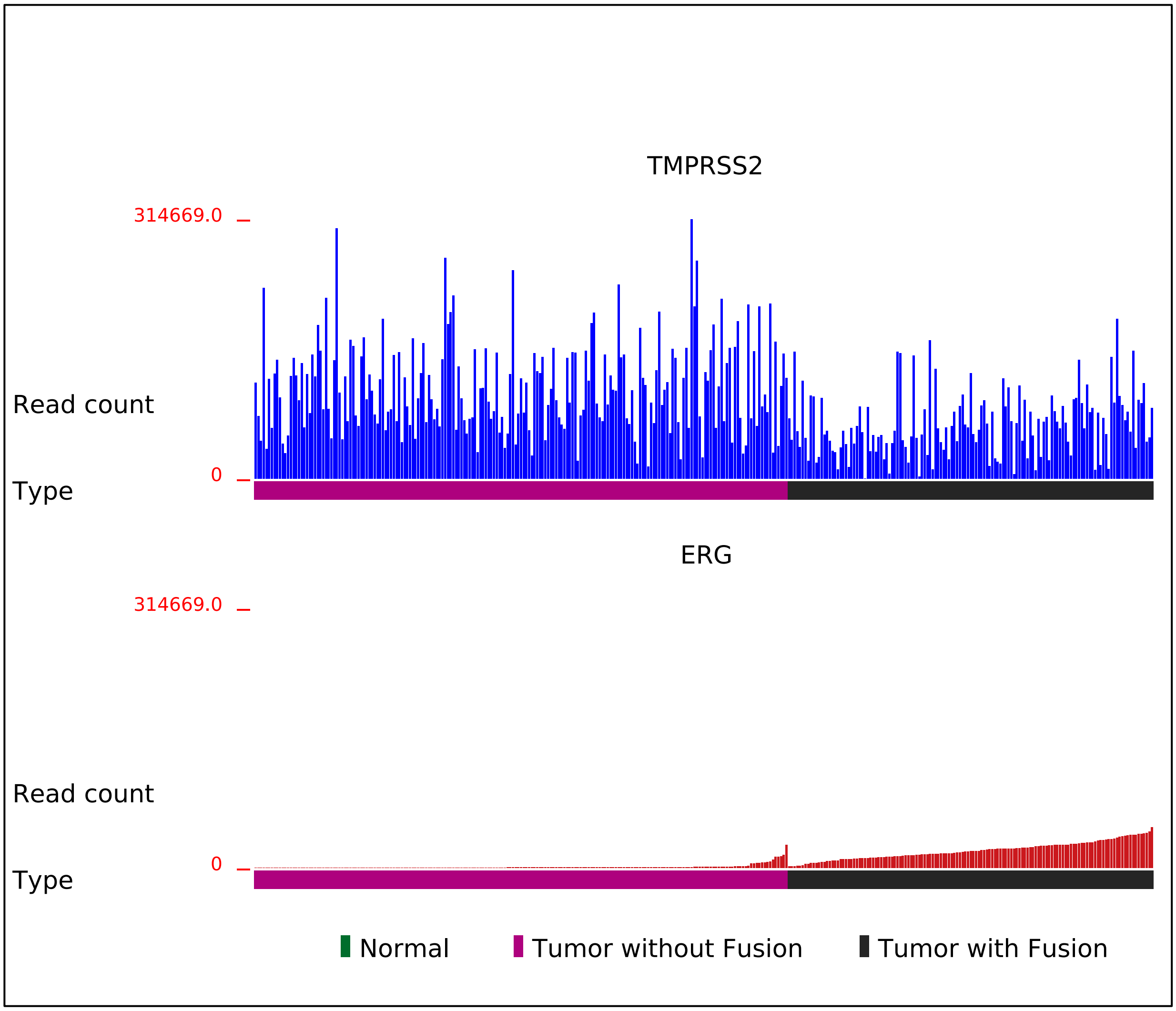


**Figure S3. Users can control the scales and other features of the gene partner expression figure.** This figure shows the same plot as Figure 2D, except that the y-axis is scaled with the same value for the top and bottom panels. Users can also designate a maximum value for the y-axes of the panels. The figure was plotted by sorting the *ERG* gene expression. This is because by default, INTEGRATE-Vis chooses the gene partner with the smallest p-value for t-statistic to cluster the type or category. Users can also control the sorting by either the 5’ or the 3’ gene.

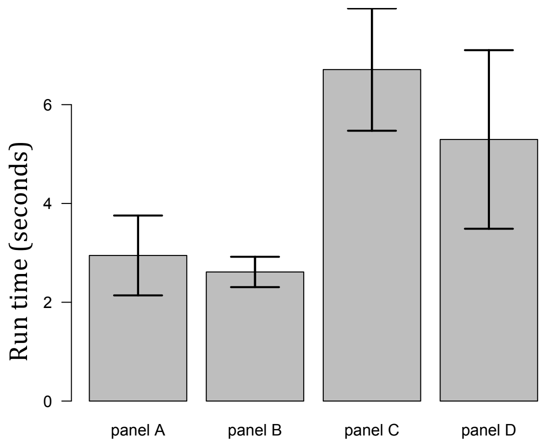


**Figure S4. INTEGRATE-Vis is very efficient.** The runtime for each type of plot as shown in Figure 2 is usually a couple of seconds. Panel C involves calculating read coverage on each nucleotide, and Panel D is run with a cohort of 333 patients. Tested using a machine with Intel Xeon CPU 2.50GHz.
